# Comparison of cryogenic and non-cryogenic droplet impact dynamics at low Weber numbers

**DOI:** 10.1038/s41598-025-90974-5

**Published:** 2025-02-25

**Authors:** Giovanni Tretola, Konstantina Vogiatzaki

**Affiliations:** Department of Engineering Science, University of Oxford, Parks Road, Oxford, OX1 3PJ UK

**Keywords:** Engineering, Mechanical engineering

## Abstract

Cryogenic fluids are crucial in applications such as rockets, cryosurgery and energy storage, where they can come in contact with surfaces. Thus, their impact dynamics are of interest. Experiments under cryogenic conditions are very expensive and not always accurate, mainly due to limitations of equipment operating in very low temperatures. Although simulation tools can provide useful insights, currently very few commercial and open-source software tailored for ultra low temperature conditions exist. In this work we present a novel numerical framework used to provide insight into the impact dynamics of cryogenic droplets with solid surfaces. Our aim is to explore whatever conclusions for droplet spreading dynamics from the current literature for droplets at non-cryogenic conditions can be applied to cryogenic droplets as well. We explore different impacting conditions, varying the initial impact velocity, of cryogenic and non-cryogenic cases, while maintaining the same Weber, Ohnesorge and Reynolds numbers between cryogenic and non-cryogenic cases. The impact on a solid surface is investigated first for a water droplet and then for a liquid oxygen droplet moving into gaseous nitrogen. For the latter, the ambient temperature and pressure are below the oxygen critical point, limiting the investigation at a sub-critical regime. The algebraic volume of fluid method with adaptive mesh refinement is employed. Numerical treatments to improve the interface description are also implemented. The simulations have been performed in OpenFOAM with a newly developed code. The results obtained are analysed both qualitatively and quantitatively, comparing the droplet morphology evolution for the two fluids. Differences are observed mainly in the receding stage, once the droplet has reached the maximum spreading, with the receding stage of the cryogenic case characterised by a faster dynamic.

## Introduction

The interest in the study of cryogenic applications has recently increased among the academic and industrial community, due to the potential use of cryogenic liquids to a range of fields including energy storage, cooling, quantum computing and even biomedical applications like cryosurgery. Interaction of cryogenic liquid droplets and jets with solid surfaces is one of the more intriguing areas within the cryogenic field that however only limited research has been performed, mostly from the experimental side. For example an interesting application involving impact dynamics is in cryosurgery, where the extreme cold produced by liquid nitrogen (LN$_2$) vaporisation is used to destroy abnormal tissues. LN$_2$ is applied directly to the cancer cells with a spraying device. Various physical phenomena occur during the impact of a non-cryogenic liquid droplet on a solid substrate, such as the spreading, fingering, air entrapment, coalescence, shedding, solidification, bouncing, and splashing. Several reviews are present in literature that classify these different phenomena (see for example^1^) using non-dimensional numbers. Physically these phenomena are mostly controlled by the fluid properties, the surface properties and the impact velocity. If the same non dimentional parameters also control the impact of cryogenic fluids has not been fully investigated yet.

Numerical simulations are fundamental to highlight the role of the different physics behind droplet impact since they can provide insight into processes that experimentation is hard. Several numerical approaches have been proposed in the literature over the years for the modelling of multiphase interfacial phenomena with non-cryogenic fluids. For example, great effort has been dedicated to correctly account for the transport of the interface and the consideration of the capillary forces. The role of the capillary forces is important both in cases with low velocity impact where capillary forces dominate throughout the process as well as to high impact case where although in the initial stages of the impact inertia is dominant as the droplets spread and slow down, capillarity becomes significant^2–4^.

Regarding cryogenic droplets impact, literature both from the numerical and the experimental side is very limited and mostly focusing on phenomena relevant to heat transfer and phase change but not impact dynamics. The evaporation process for single liquid nitrogen droplets when submerged into a non–cryogenic liquid has been studied by Rebelo et al.^5^, showing the influence of the initial droplet size and of the surface tension on the evaporation rate. Van Limbeek et al.^6^ studied experimentally the impact of a single liquid nitrogen drop on a smooth sapphire prism through high-speed frustrated total internal reflection imaging. Changing the prism temperature and drops impact velocity a phase diagram of the impact characteristics was observed. They showed how also in these conditions the cooling power of a drop is strongly related to the wetting behaviour of the impacting drops.

In this investigation, we focus on the spreading behaviour of cryogenic droplets, providing novel insight into whether the current literature established for non-cryogenic conditions can be applied to the case of cryogenic drops. In this regards, different impacting conditions are applied to a cryogenic and non–cryogenic cases, characterised by the same Weber (*We*), Ohnesorge (*Oh*) and Reynolds (*Re*) numbers. The influence of the impact conditions on both the cryogenic and non cryogenic frameworks is investigated varying the initial impact velocity. To the authors’ knowledge, this is the first numerical investigation focusing on the simulation of the impact dynamics of cryogenic droplets and how they differ in comparison to a non cryogenic case droplet impact. First, the impact of a water droplet on a flat, solid surface is investigated. Then, the impact behaviour onto a solid surface of a liquid oxygen ($\text{ LO}_2$) droplet moving into gaseous $\text{ N}_2$ is simulated at the same conditions. The algebraic volume of fluid (VoF) method is employed with a new adaptive mesh refinement technique on the interface region. Numerical treatments to improve the interface description are implemented as well. The simulations have been performed in OpenFOAM with a newly developed code tailored for liquid/solid interaction. The results obtained are analysed both from a qualitative and quantitative point of view.

## Methods

**Fig. 1:**
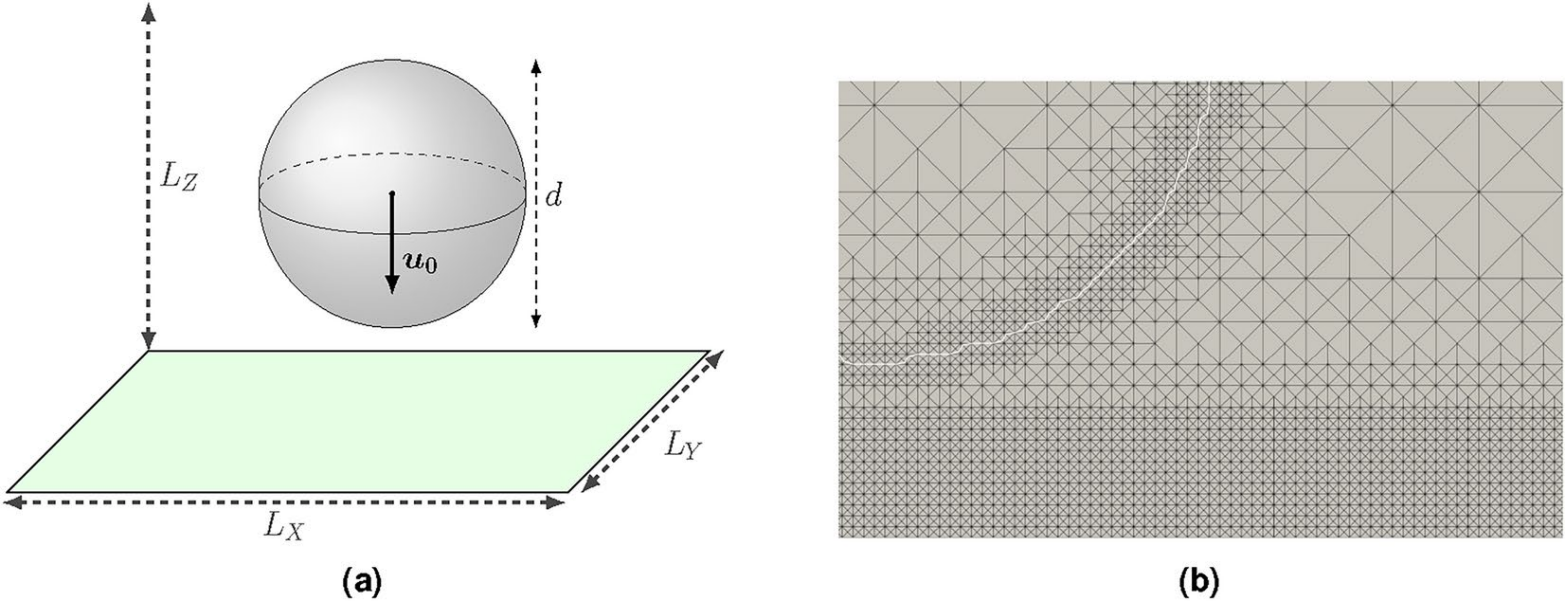
(**a**) Initial droplet configuration and numerical domain. (**b**) Snapshots of the adaptive mesh refinement: the snapshot is taken before the impact, where most of the refinement is concentrated around the interface and at the wall.

**Table 1:** Transport properties and operating condition investigated.

Cases	$\text{ LO}_2\text{-N }_2$				Water–air				Nondimensional parameters		
	$d_{0}$ (mm)	$u_{0}$ (m/s)	$\rho _l/\rho _g$	$\mu _l/\mu _g$	$d_{0}$ (mm)	$u_{0}$ (m/s)	$\rho _l/\rho _g$	$\mu _l/\mu _g$	*We*	*Re*	*Oh*
1	0.9465	0.567	438	47.3	2.05	1	847	46	28.38	2400	$2.2\times 10^{-3}$
2	0.9465	1.134	438	47.3	2.05	2	847	46	113.52	4800	$2.2\times 10^{-3}$
3	0.9465	2.268	438	47.3	2.05	4	847	46	454.08	9600	$2.2\times 10^{-3}$

The details of the numerical methodology adopted in this study are presented in the supplementary material. In the supplementary material more details are presented: in Sec. 1 the governing equations, in Sec. 2 the numerical framework for the interface treatment and in Sec. 3 the adaptive mesh refinement strategy. Here only the main points of the methodology and the set-up employed are included. Our suggested framework is designed for simulations of compressible and immiscible fluids. The interface tracking of the two-phase flow is performed with the Volume of Fluid (VoF) method as basis using the Open Source code OpenFOAM^7^. The native solver *compressibleInterFOAM* was used as starting point and modifications were made to account for the interface treatment. More details about the implementation into the method and the influence of these modifications, along with the validation of the implemented solver, can be found in previous studies^8,9^.

The impact conditions are defined in terms of Weber and Reynolds numbers. The first non dimensional number represents the ratio between the fluid inertia and the surface tension force and is defined as $We = \rho _l u_{0}^2 d_{0} /\sigma$, where $u_{0}$ is the initial impact velocity, $d_{0}$ the initial droplet diameter, $\rho _l$ the liquid density and $\sigma$ the surface tension. The Reynolds number represents the ratio of inertial forces to viscous forces, is defined as $Re = \rho _l u_{0} d_{0} /\mu _l$, where $\mu _l$ is the liquid dynamic viscosity. The numerical domain and initial configuration is shown in Fig. 1a. The domain has an extension of $L_{X} = L_{Y} = 4 \times d$ and $L_{Z} = 2.5 \times d$. On the x and y direction 160 cells are used, while 100 on the z direction. A level of refinement of 7 is employed, which means that the cell is halved up to 7 times. The refinement is based on the following variables:

- gradient of volume fraction $\alpha$;
- vorticity magnitude;
- distance from the solid surface;

and when the first two are above a certain threshold, and the last one below, the cell is refined. An example of a section of the refined grid is shown in Fig. 1b. In the supplementary material is shown a comparison between a case where only the interface is refined versus the refinement strategy here reported.

At the initial time step, the spherical droplet is set $0.1 \times d_0$ away from the solid wall. The velocity field within the droplet is initialised with the initial velocity of the moving droplet $u_0$. The top and lateral boundaries are treated as open boundaries, while on the bottom boundary the no-slip boundary condition is applied for the velocity fields, while a Neumann boundary condition is satisfied for the other fields. Applying this condition for the volume fraction means that we neglect the influence of the contact angle on the boundary, as the focus of this study is only on the influence of the cryogenic fluids on the impact behaviour. Moreover, exact values or guidelines for cryogenic contact angles do not currently exist. In a future study a sensitivity analysis of the contact angle influence will be performed.

The conditions investigated are summarised in Table 1. The cryogenic case and the non–cryogenic case have the same non dimensional parameters, characterised by the *We*, *Re* and *Oh*. Although the non dimensional parameters are constant, at cryogenic conditions, the gaseous phase is characterised by lower viscosity and higher density compared to the non–cryogenic case and the surface tension is lower.

**Fig. 2:**
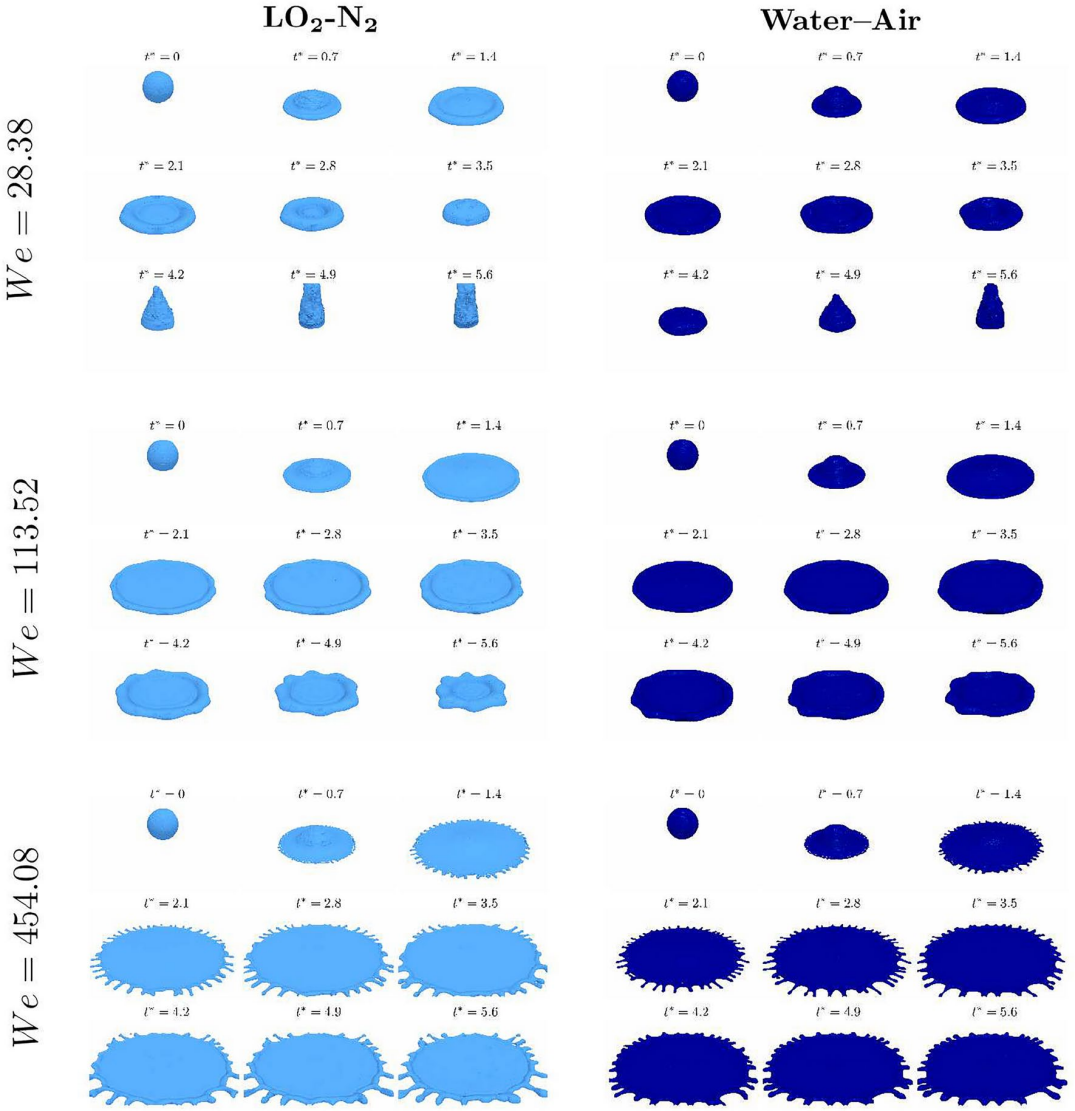
Evolution of the $\alpha = 0.5$ iso-surface for the different cases. Left column: cryogenic case; right column: non–cryogenic case. From top to bottom: increases the impact *We* number. The different time steps are observed every $t^* = 0.7$.

## Results

The operating point investigated falls into the rebound regime (based on the categorisation of non-cryogenic fluids). Figure 2 shows the evolution over time of the droplet as the iso-surface $\alpha = 0.5$. The initial dynamics of the spreading are similar for the two cases and follow a non-splashing regime, as expected at this operating point^10^. At the initial stages, for both cases the droplet is forming a circular crown at the side. Once the maximum spreading diameter has been reached, differences start arising between the cases: for the non-cryogenic case the droplet starts receding keeping a disk shape, until it reaches the centre of the disk where a liquid jets formation is observed. For the cryogenic case, the shape is less regular and the jet formation at the centre happens earlier than the non–cryogenic case. For both the cryogenic and non cryogenic cases, the increase of the initial impact velocity results into a further spreading.

**Fig. 3:**
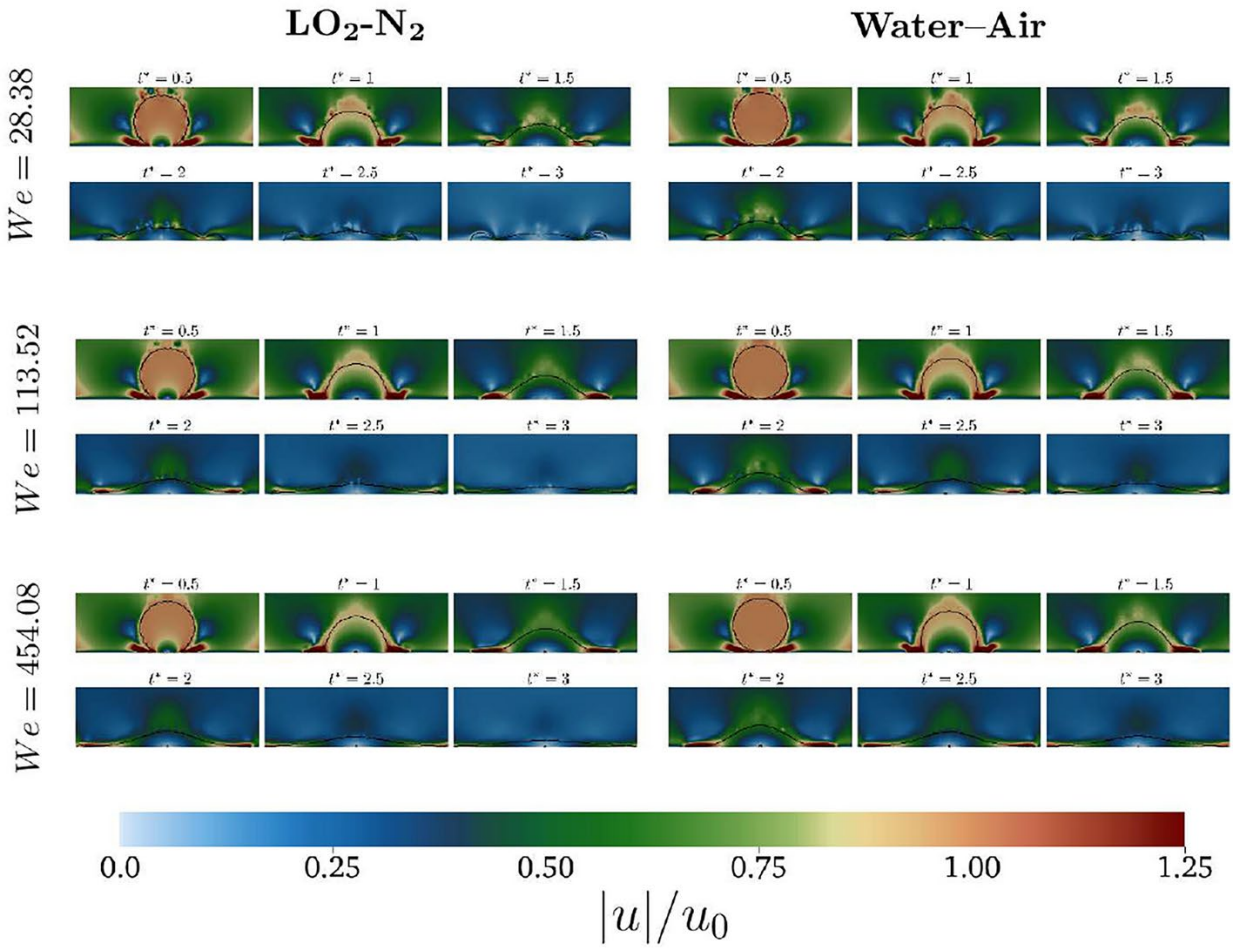
Focus on the differences at the interface during the impact. Left column: cryogenic case; right column: non–cryogenic case.

Figure 3 shows a zoom in to the droplet edge during the spreading for the cryogenic and non-cryogenic cases, for two different impact conditions. More instabilities are observed at the edge of the rim when *We* is increased up to 454.08. The formations of fingers is observed for both the cryogenic and non cryogenic impact while the droplet still spreads. The fact that more instabilities are observed for the cryogenic case is due to the lower value of surface tension characterising the cryogenic fluids, although the *Oh* number is kept constant between the cases (see Table 1).

**Fig. 4:**
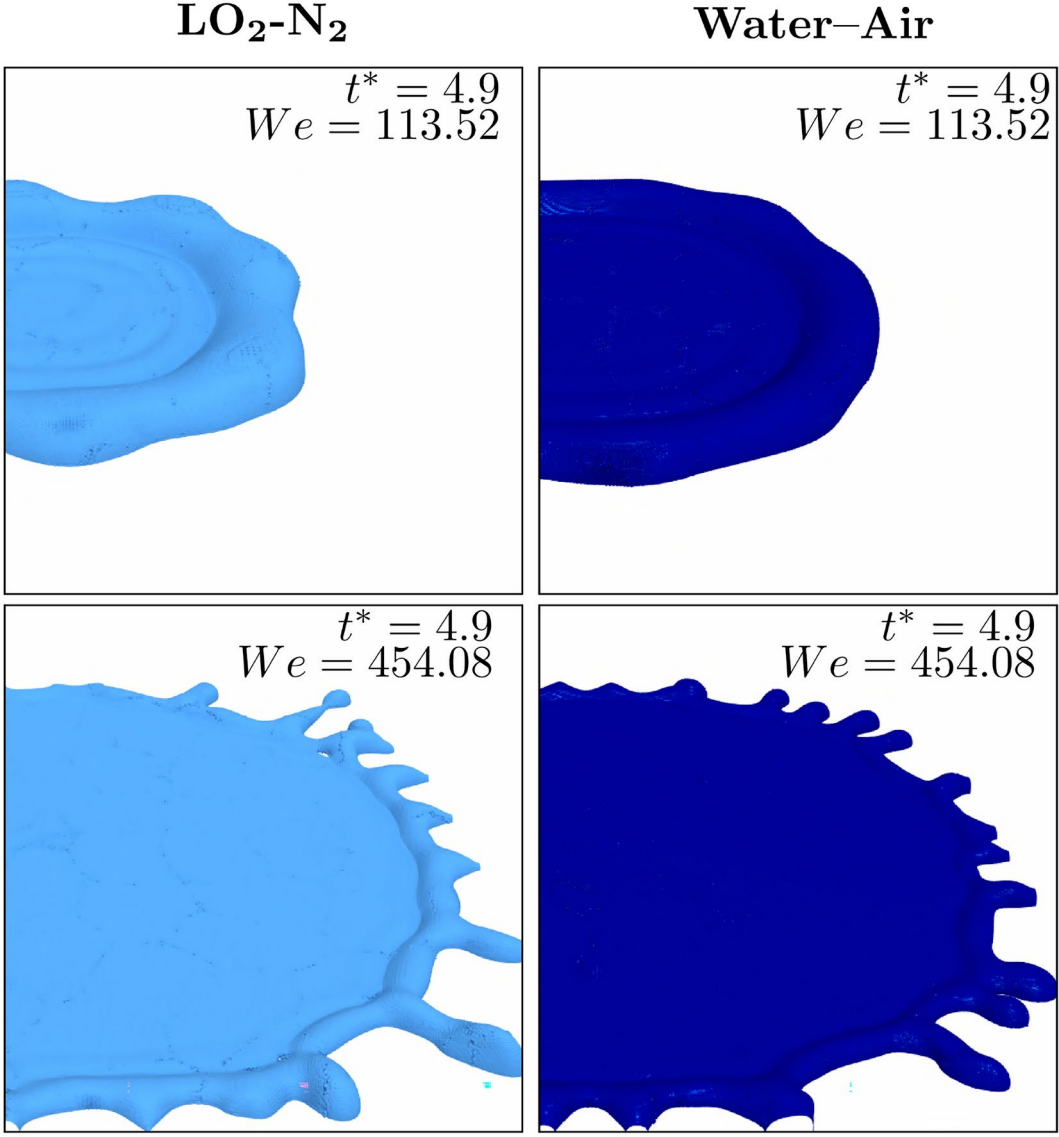
Velocity distribution at the mid section. For each case, the velocity is normalised by the corresponding impact velocity  $u_{0}$, see Table 1.

To analyse the source of these differences, the velocity distribution (normalised with the corresponding impact velocity $u_{0}$, see Table 1) at the mid section at different time steps is shown in Fig. 4. The spreading is very similar for both cases, with the velocity peak of $|u| > u_0$ present at the lamella extending at the wall. Once the maximum spreading is reached, the cryogenic case recedes faster. For the lower *We* cases (first column), the edges are larger for the cryogenic case and when the droplets recedes to the centre, resulting into a large blob with higher velocity at the tip. The non–cryogenic case instead presents a higher velocity in the gaseous phase, right above the droplet, which emerges from the receding in an elongated shape. Increasing the *We*, the recoil is not observed yet and the lamella velocity decreases after the first impact. When the non cryogenic fluids are considered (second row), the velocity are higher during the spreading.

**Fig. 5:**
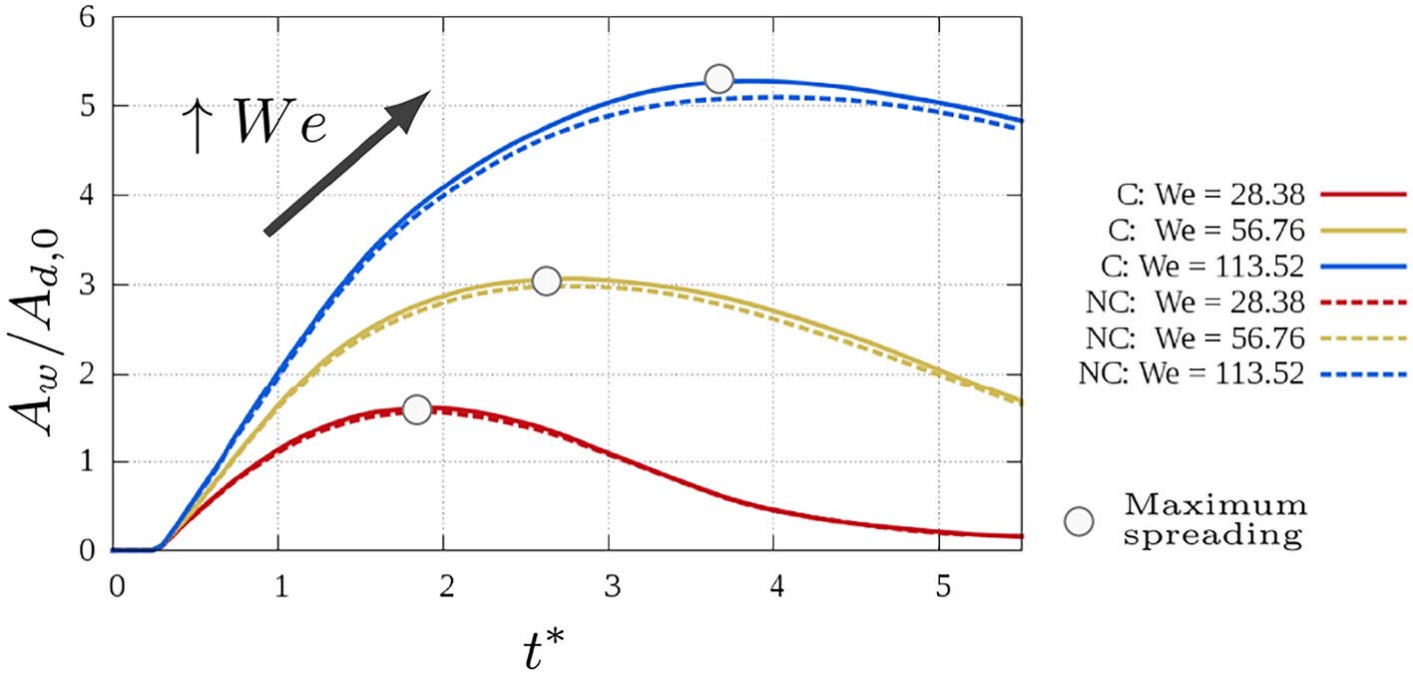
Wet area over time for the different cases. The grey circles indicate the instant when the maximum spreading is reached.

Figure 5 shows the wet surface area at the wall $A_w$ over the time for the cryogenic (C) and non–cryogenic (NC) cases. The area is normalised by the initial droplet surface $A_{d,0}$. In non-dimensional terms, the wet area is identical for the non-cryogenic and the cryogenic cases, fixing *We* and *Re*. Increasing the impact velocity, the wet area increases as well, and the peak value is reached at a later time. Furthermore, when absolute time is considered, once the peak is reached, the cryogenic case shows a faster decay than the non–cryogenic.

A further analysis of the morphology is obtained through the centre of mass and the mass velocity of the droplet, defined as:

- Centre of mass of the droplet ${\mathscr {C}}_d$:
  $${\mathscr {C}}_d = \left( {\mathscr {C}}_{d,x},{\mathscr {C}}_{d,y},{\mathscr {C}}_{d,z}\right) = \frac{\int _{V} \alpha \textbf{x}dx }{\int _V \alpha dx }$$
- Droplet velocity $$\textbf{u}_d$$:
  $$\textbf{u}_d = \left( u_{d,x},u_{d,y},u_{d,z}\right) = \frac{\int _{V} \alpha \textbf{u}dx }{\int _V \alpha dx }$$

**Fig. 6:**
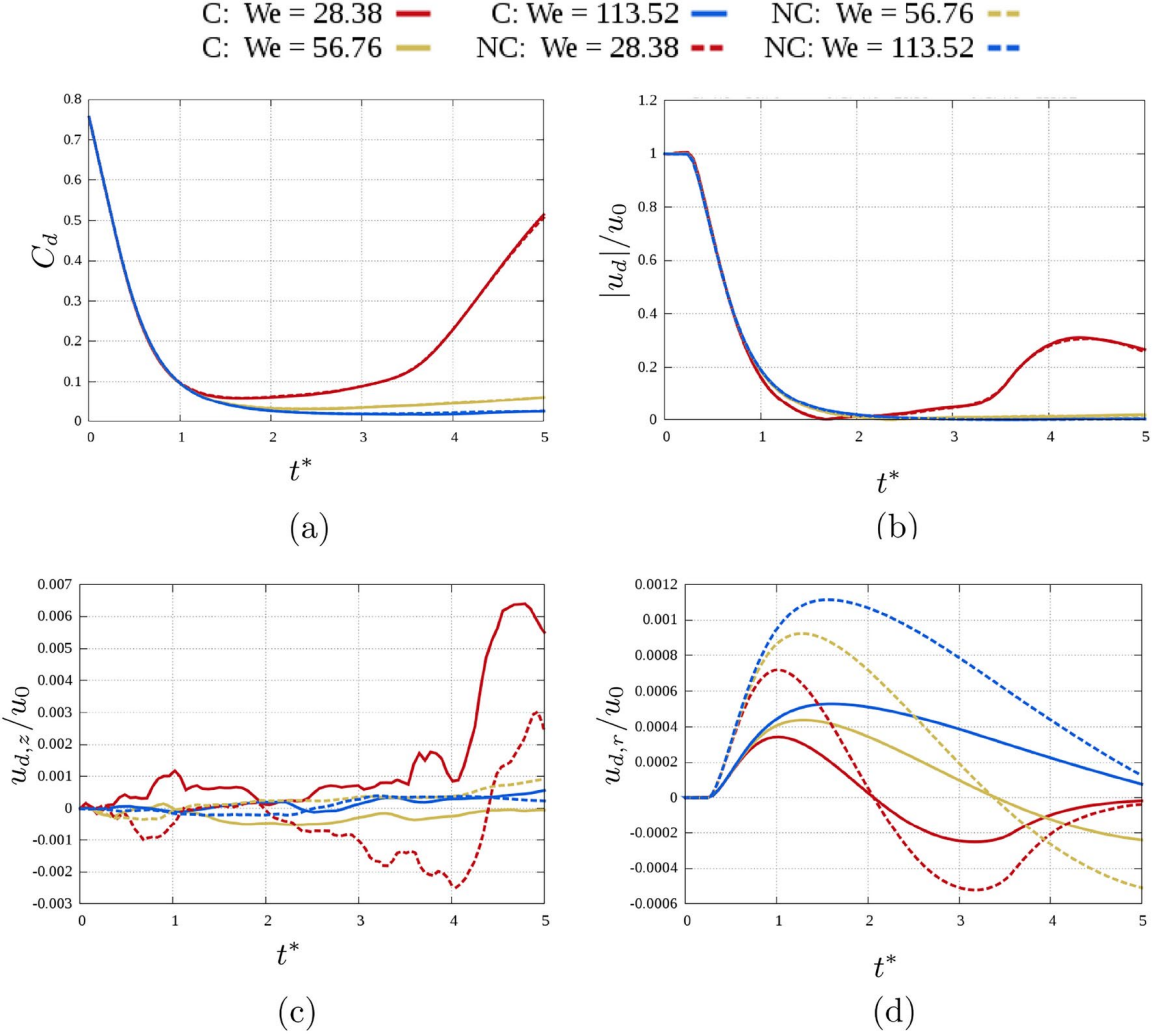
Comparison of profile over time between the different cases of: (**a**) centre of mass, (**b**) droplet velocity magnitude, (**c**) vertical component droplet velocity, (**d**) radial component droplet velocity.

Figure 6 shows the evolution trend over time of the droplet centre of mass and velocity magnitude and components for the different cases.

Looking at the centre of mass, an increase after reaching the minimum is observed only for $We = 28.38$(being the only case when rebound is observed). For the same reason the same trend is observed for the droplet velocity magnitude $|u_d|$. The main differences, both increasing $u_{0}$ and moving from cryogenic and non cryogenic cases, are observed for the radial component $u_{d,r}$. Increasing *We*, the maximum value reached increases as well, with the inversion of sign not reached when $We = 113.52$. Furthermore, the radial velocity is higher for the non cryogenic case.

**Fig. 7:**
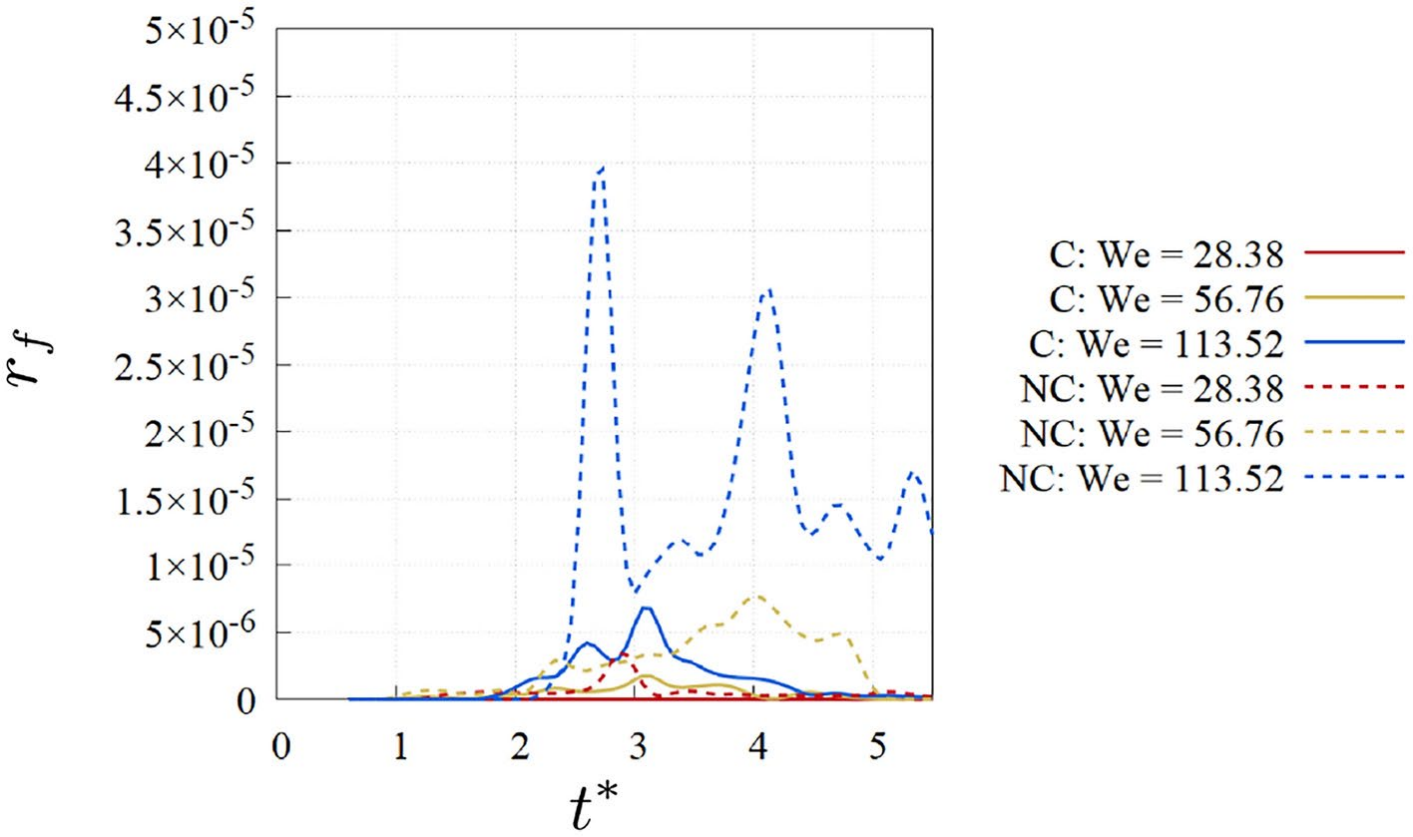
Ratio between viscous and capillary forces on the interface over time for the different cases.

The ratio between the viscous and the capillary forces on the interface is defined as $r_{f} = |\textbf{f}_{\mu ,I}/ \textbf{f}_\sigma |$, where $\textbf{f}_{\mu ,I} = \tau \cdot \textbf{n}_I$ represents the viscous force on the interface. This ratio is integrated over the interface area, to represent the global ratio, and its trend over time is showed in Fig. 7 for the different cases.

For both the cryogenic and non cryogenic cases the value is well below 1, which is the threshold of the viscous forces over the capillary ones. This is in agreement with the rebound regime observed for all the cases. Nevertheless, the absolute values, at the same *We*, are higher for the non cryogenic cases. This explains the different interface deformation observed in Fig. 2.

## Discussion

In this study, the spreading behaviour for cryogenic droplets onto solid surfaces is reported using numerical simulations. Furthermore, the influence of the We and Re numbers on the impact is analysed and is compared to the influence of the same parameters for non cryogenic conditions. Heat transfer is not considered, focusing only on the morphology of the impact. First the shape of liquid droplet during the impact is analysed. The differences between the cases arise during the receding stage, where the cryogenic droplet recedes faster towards the centre than the non–cryogenic one. As a result, a larger blob is rising after the liquid edge reaches the centre of the droplet. Although in non–dimensional terms the receding times coincided, in absolute term the receding stage is slower for the non–cryogenic droplet, which means that the liquid keeps in contact with the surface longer. From an application point of view, this can make a considerable difference, for example in cryo–surgery as this implies a higher contact time with the infected tissue. To quantify the impact, the comparison then focused on the wet area $A_w$ and on the droplet shape parameters as the centre of mass and velocity, ${\mathscr {C}}_d$ and $|u_d|$. In non-dimensional terms, the spreading is identical for the non-cryogenic and the cryogenic cases, fixing *We* and *Re*. It is worth noting that, when absolute time is considered, once the peak is reached, the cryogenic case shows a faster decay than the non–cryogenic. When the morphology of the droplet is considered, the lower surface tension for the cryogenic cases results in a greater instability of the interface, despite the same *Oh*. This difference results in the receding stage, once the initial kinetic energy of the droplet is dissipated. This can be due to the gaseous phase, as at cryogenic conditions the gaseous phases are characterised by lower viscosity and higher density compared to non–cryogenic conditions. In future investigations a wide range of operating conditions will be studied, introducing also various contact angles. Nevertheless, it is not clear if existent models of dynamic contact angles can be used for cryogenic fluids and thus further research is required.

## Supplementary Information

Supplementary Information.

## Data Availability

The datasets analysed during the current study is available from the corresponding author on reasonable request.
